# Elucidation of binding preferences of YEATS domains to site-specific acetylated nucleosome core particles

**DOI:** 10.1016/j.jbc.2022.102164

**Published:** 2022-06-19

**Authors:** Masaki Kikuchi, Satoshi Morita, Mie Goto, Masatoshi Wakamori, Kazushige Katsura, Kazuharu Hanada, Mikako Shirouzu, Takashi Umehara

**Affiliations:** 1 RIKEN Center for Biosystems Dynamics Research, Tsurumi, Yokohama, Japan; 2 PRESTO, Japan Science and Technology Agency (JST), Kawaguchi, Saitama, Japan

**Keywords:** acetyllysine, chromatin, crystal structure, epigenetics, nucleosome, transcription, YEATS domain, AG, Azami-Green, BD, bromodomain, cDNA, complementary DNA, HDAC6, histone deacetylase 6, ITC, isothermal titration calorimetry, Kac, acetylated lysine(s), KDAC, lysine deacetylases, NCP, nucleosome core particle, PTM, posttranslational modification, TSA, trichostatin A, YEATS, Yaf9, ENL, AF9, Taf14, and Sas5

## Abstract

Acetylated lysine residues (Kac) in histones are recognized by epigenetic reader proteins, such as Yaf9, ENL, AF9, Taf14, and Sas5 (YEATS) domain-containing proteins. Human YEATS domains bind to the acetylated N-terminal tail of histone H3; however, their Kac-binding preferences at the level of the nucleosome are unknown. Through genetic code reprogramming, here, we established a nucleosome core particle (NCP) array containing histones that were acetylated at specific residues and used it to compare the Kac-binding preferences of human YEATS domains. We found that AF9-YEATS showed basal binding to the unmodified NCP and that it bound stronger to the NCP containing a single acetylation at one of K4, K9, K14, or K27 of H3, or to histone H4 multi-acetylated between K5 and K16. Crystal structures of AF9-YEATS in complex with an H4 peptide diacetylated either at K5/K8 or K8/K12 revealed that the aromatic cage of the YEATS domain recognized the acetylated K8 residue. Interestingly, E57 and D103 of AF9, both located outside of the aromatic cage, were shown to interact with acetylated K5 and K12 of H4, respectively, consistent with the increase in AF9-YEATS binding to the H4K8-acetylated NCP upon additional acetylation at K5 or K12. Finally, we show that a mutation of E57 to alanine in AF9-YEATS reduced the binding affinity for H4 multiacetylated NCPs containing H4K5ac. Our data suggest that the Kac-binding affinity of AF9-YEATS increases additively with the number of Kac in the histone tail.

Posttranslational modifications (PTMs), such as acetylation and methylation of lysine residues, occur in a wide variety of cellular proteins, including core histones (1, 2, 3). These PTMs of the N-terminal tails of the core histones H2A, H2B, H3, and H4 can be inherited across cell divisions through active maintenance by lysine acetyltransferases and methyltransferases and control chromatin structure and gene expression in eukaryotes (4, 5, 6). These PTMs are added and maintained in the nucleosome core particle (NCP), a chromatin compaction unit formed by wrapping the histone octamer (two copies of each of the four core histones) with a 145 to 147 bp dsDNA (7, 8, 9). In the NCP, the N-terminal tails of the core histones protrude through the DNA (7) and are accessible to a variety of chromatin-associated factors, such as ‘writers’, ‘readers’, and ‘erasers’ of the PTMs (5).

Lysine acetylation of H3 and H4 is a key regulator of gene expression; its effect can be direct or indirect. The direct effect is caused by the removal of the positive charges of lysine side chains by acetylation. Consequently, the acetylation of the N-terminal tail of H4 decreases its affinity to nucleosome-length DNA (10). Single acetylation of H4 at K16 in nucleosomes reduces the internucleosome interaction, and multiple acetylation of H4 at K5/K8/K12/K16 (*i.e.*, hyperacetylation of H4) causes internucleosomal decompaction in reconstituted systems (11, 12, 13), suggesting that lysine acetylation in the nucleosome decompacts the higher-order chromatin structure even in the absence of chromatin-associated factors. The indirect effect of lysine acetylation on chromatin regulation is mediated by recruitment of a ‘reader’ domain (14, 15). Acetylated lysine (Kac) in the N-terminal tail of H3 or H4 provides a binding scaffold for reader proteins such as those containing a bromodomain or Yaf9, ENL, AF9, Taf14, and Sas5 (YEATS) domain. The bromodomain was the first Kac-binding domain to be identified; it consists of approximately 110 residues and forms a four-α-helix bundle structure (16, 17, 18). In humans, 61 bromodomains are present in 46 different proteins (18). Another Kac-binding domain, the YEATS domain, consists of approximately 130 residues and forms an antiparallel β-sheet structure that can recognize and bind Kac and some acylated lysines (19, 20, 21, 22, 23, 24, 25, 26, 27, 28). In humans, four YEATS domains are present in four different proteins (AF9, ENL, YEATS2, and GAS41) (29).

Lysine acetylation in the histone tail enhances the binding of the bromodomain and the YEATS domain, as demonstrated by biochemical analyses performed mostly with acetylated peptides as binding substrates (21, 26, 27, 28, 30, 31, 32), but rarely with acetylated nucleosomes (33). Arrays of histone tail peptides containing a variety of combinatorial PTMs, immobilized on a membrane, beads, or a plate, have been developed for such analyses (34, 35, 36). They are especially useful for validation of antibodies recognizing a PTM in a histone tail. However, it is better to validate the preference of chromatin-associated factors for PTMs by using nucleosomes with PTMs as substrates, because a histone peptide and a nucleosome differ chemically and physically, and the latter better reflects the chromatin environment.

Binding of YEATS domains to acetylated histones has been analyzed mostly using acetylated histone peptides; for example, binding of AF9-YEATS to an H3K9ac peptide (21) and binding of the YEATS domains of ENL, YEATS2, and GAS41 to an H3K27ac peptide (26, 27, 28, 31, 32, 37) have been analyzed by isothermal titration calorimetry (ITC) and nuclear magnetic resonance. On the other hand, there is only one report that AF9-YEATS binds to unmodified and H3K9-acetylated NCPs in a native gel shift assay (38). Colocalization of ENL, YEATS2, and GAS41 with H3K27ac in cells has been analyzed by ChIP-seq, co-IP, or both (26, 27, 31, 32). However, the binding affinity and selectivity of each YEATS domain for binding to unmodified and specifically acetylated nucleosomes have not been determined.

Currently, nucleosomes with residue-specific acetylation(s) are prepared by native chemical ligation or by genetic code reprogramming. Studies on Kac incorporation into histones through genetic code reprogramming are still limited (39, 40, 41, 42). Chin *et al*. (42) reported biochemical preparation of histones H2A, H2B, and H3, each of which carried genetically installed Kac at a single defined residue. We have developed a biochemical methodology to synthesize a protein in which Kac, or a variety of lysine analogs, can be introduced at multiple positions through reprogramming of the genetic code reprogramming and engineering of the translation termination system (13, 43, 44, 45). In particular, utilization of a cell-free protein synthesis system enables milligram-scale production of the Kac-containing H4 protein (13). An NCP-containing H4 tetra-acetylated at K5/K8/K12/K16 produced by this methodology has essentially the same structure and stability as the unmodified NCP (13), demonstrating its structural integrity. However, the genetic preparation of a variety of Kac-histones and NCPs for comparative biochemical analyses has yet to be performed.

Here, we reconstituted NCPs containing a series of residue-specific acetyllysine(s) in the N-terminal tail of histone H3 or H4 for comparative validation of Kac-binding proteins. We prepared a mini library of Kac-containing NCPs immobilized on an avidin-coated multiwell plate and optimized the binding conditions using several bromodomain proteins. We quantitatively compared the Kac-binding preferences of four human YEATS domains toward acetylated nucleosome or acetylated tail peptides and found that AF9-YEATS binds to unmodified NCP and that AF9 and two other YEATS domains bind to the NCP containing H4 tetra-acetylated at K5/K8/K12/K16. To understand how the AF9-YEATS domain recognizes multiacetylation of H4, we solved its crystal structures in complex with H4 peptides diacetylated either at K5/K8 or K8/K12. In both structures, AF9 recognized K8ac at the aromatic cage of the YEATS domain, while residues outside the aromatic cage interacted with K5ac or K12ac. We discuss the structural mechanism of the recognition of H4 multiacetylation by AF9-YEATS and a potential advantage of the NCP library over the conventional histone peptide array in characterizing Kac-binding proteins.

## Results

### Preparation of an acetylated NCP array

**Figure 1:**
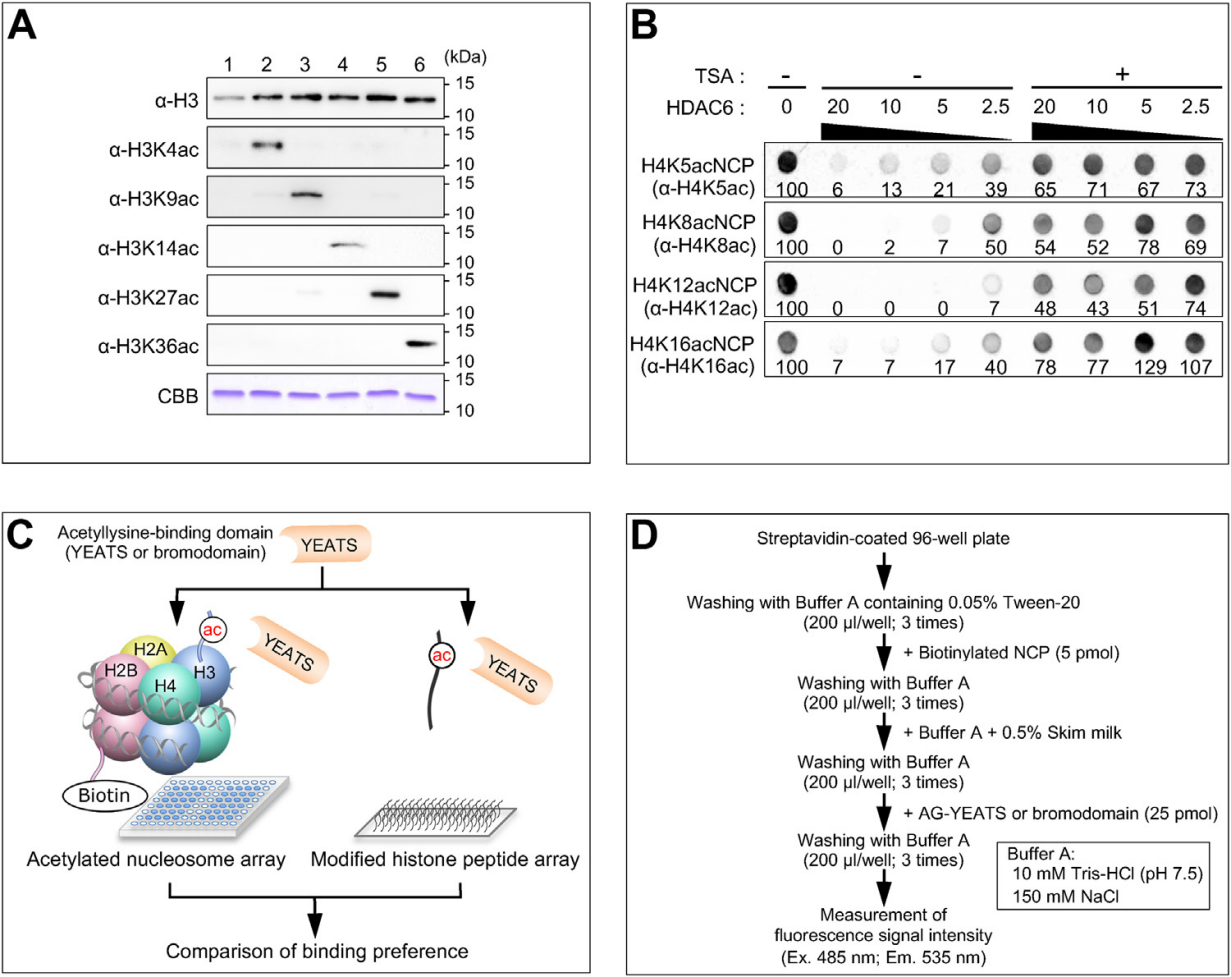
**Development of a library of nucleosome core particles (NCPs) containing specific acetylated lysine residue(s).***A*, synthesis of specifically acetylated histone H3 proteins. Western blots using antibodies recognizing the indicated histone species. Lane 1: unmodified H3; lane 2: K4-acetylated H3; lane 3: K9-acetylated H3; lane 4: K14-acetylated H3; lane 5: K27-acetylated H3; and lane 6: K36-acetylated H3. *B*, deacetylation of H4-acetylated NCPs by HDAC6. The positions of the introduced mono-acetylation and the antibody recognition residue are shown on the *left*. In the dot blot analysis, 10 ng/μl of the Kac-containing NCP was incubated with the indicated concentrations (ng/μl) of HDAC6 in the presence (+) or absence (-) of 1 μM trichostatin A (TSA). Signal intensity (%) is shown below each dot. *C*, schematic representation of the NCP-based and peptide-based binding assays. *D*, outline of the NCP-based binding assay. The excitation wavelength (Ex.) is 485 nm and the emission wavelength (Em.) is 535 nm. CBB, histone H3 proteins in a Coomassie Brilliant Blue-stained SDS-PAGE gel; HDAC6, histone deacetylase 6; Kac, acetylated lysine residue; NCP, nucleosome core particle.

To evaluate the binding preferences for position-specific Kac in various Kac readers, we synthesized Kac-containing histones H3 and H4 at the milligram scale. We used the transcription–translation-coupled, *Escherichia coli* cell-free protein synthesis system with a reprogrammed genetic code and an engineered translation termination system (13, 43). We prepared a series of histones H3 and H4 containing Kac at the specified position(s) known to be acetylated in humans (46). We prepared seven kinds of Kac-containing H4 (*i.e.*, K5ac, K8ac, K12ac, K16ac, K5/K8-diacetylated, K5/K12-diacetylated, and K5/K8/K12/K16-tetra-acetylated) (13) and five kinds of Kac-containing H3 (*i.e.*, K4ac, K9ac, K14ac, K27ac, and K36ac). Selective acetylations of the designed residue(s) of H3 were confirmed by Western blotting with a series of antibodies, each recognizing respective Kac (Fig. 1*A*).

First, we examined whether the reconstituted NCP with Kac is functional as a substrate for lysine deacetylases (KDACs) *in vitro*. We tested activity of histone deacetylase 6 (HDAC6) toward NCP-containing H4 with K5ac, K8ac, K12ac, or K16ac. Our dot blot analysis showed that HDAC6 deacetylated all four Kac residues (Fig. 1*B*). Subsequently, we examined whether a HDAC-specific inhibitor, trichostatin A (TSA), inhibits deacetylation of Kac-containing NCPs. TSA (1 μM) inhibited deacetylation of all four Kac residues by HDAC6 (Fig. 1*B*). HDAC6 seemed to prefer K12ac in a dose-dependent assay with NCPs acetylated at single H4 residues. These results indicate that reconstituted NCPs with residue-specific acetylation(s) are functional substrates for a KDAC and that this acetylation is completely or almost completely erasable by a KDAC *in vitro*.

Next, to compare the binding preferences of Kac-binding proteins at the nucleosome level, we immobilized 12 kinds of Kac-containing NCPs with biotinylated H2B onto a streptavidin-coated multiwell plate (Fig. 1*C*). Next, we optimized the conditions of *in vitro* association between the reconstituted NCP and Kac-binding proteins by using three human bromodomains with known binding specificities for acetylated histone tail peptides (Fig. 1*D*). To detect binding by fluorescence intensity, the second bromodomain (BD2) of PB1, bromodomain of BAZ2B, or double bromodomain (BD1+BD2) of TAF1L was fused to a green fluorescent protein, Azami-Green (AG) (47). The binding experiments were performed in 10 mM Tris-HCl (pH 7.5) containing 150 mM NaCl (Fig. 1*D*), which is considered a physiological saline condition. Under the washing and binding conditions shown in Figure 1*D*, the AG protein alone (negative control) did not bind to any of the NCPs (Fig. S1*A*). AG-fused PB1-BD2 significantly bound to the H3K14-acetylated NCP in comparison with the unmodified NCP (*p* < 0.01; Fig. S1*A*), as expected from previous NMR and biochemical binding analyses (30, 48). BAZ2B bromodomain also preferentially bound to the H3K14-acetylated NCP (*p* < 0.01; Fig. S1*A*), which is consistent with previous reports (30, 49). The TAF1L bromodomain binds weakly to H3 or H4 acetylated at various residues, such as H3K4ac and H4K5ac (30). In the NCP array, TAF1L bound to many kinds of acetylated NCPs (Fig. S1*A*). Because the binding preferences obtained in this assay were consistent with previous reports, we concluded that the acetylated NCP-based assay established in this study is valid for evaluating the interactions of epigenetic reader proteins with posttranslationally modified nucleosomes. The binding preferences of other typical bromodomains were determined under the same assay conditions (Fig. S1*B*).

### Analysis of binding between the acetylated NCP array and YEATS domains

**Figure 2:**
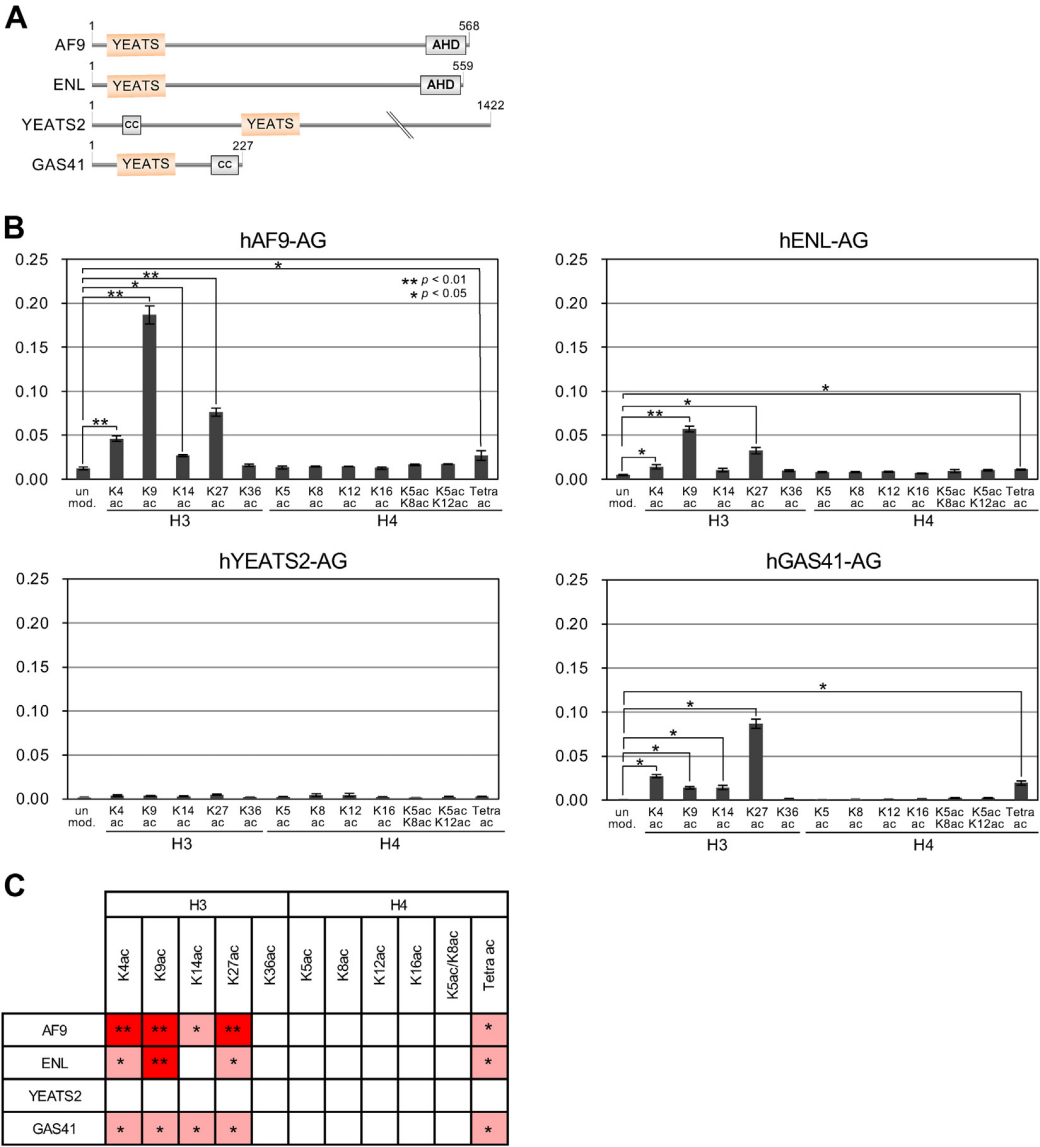
**Binding of AG-fused human YEATS domains to acetylated NCPs.***A*, domain architecture of human YEATS domain-containing proteins. *B*, binding ratios of AF9-YEATS, ENL-YEATS, YEATS2-YEATS, and GAS41-YEATS. The *x*-axis indicates histone acetylation (unmod., unmodified; Tetra ac, H4K5ac/K8ac/K12ac/K16ac). The *y*-axis indicates the binding ratio (bound fraction per input). Means ± SE (n = 3). *C*, summary of the preferences of the YEATS domains to acetylated NCPs. Two-tailed *p* values are indicated: ∗∗*p* < 0.01; ∗*p* < 0.05 (two-tailed Student’s *t* test). AHD, ANC1 homology domain; AG, Azami Green; cc, coiled-coil regions; NCP, nucleosome core particle; YEATS, Yaf9, ENL, AF9, Taf14, and Sas5.

The domain architecture of the YEATS domain-containing proteins is shown in Figure 2*A*. We purified AG-fused YEATS domains of AF9, ENL, YEATS2, and GAS41 and measured their binding to acetylated NCPs (Fig. 2*B*). In comparison with binding to the unmodified NCP, the AF9-YEATS domain bound strongly and significantly to the H3K9-acetylated NCP (*p* < 0.01), as expected from (21); it also bound significantly to the H3K4- and H3K27-acetylated NCPs (*p* < 0.01). It bound weakly to all other NCPs including the unmodified one. The ENL-YEATS domain bound significantly to the H3K4-, H3K9-, and H3K27-acetylated NCPs (*p* < 0.05) and also showed basal-level binding to all other NCPs, but it was weaker than that of AF9-YEATS.

The YEATS2-YEATS domain reportedly prefers H3K27ac (31) and binds to K27ac-containing H3 peptides with a K_D_ of 226 μM (50) or 50 μM (31). In our assay, it did not show significant interaction with any of the acetylated NCPs, although it slightly preferred H3K27ac (Fig. 2*B*). Using ITC analysis, we also found that YEATS2-YEATS protein did not bind to the K27ac-containing H3 (15–39) peptide (Fig. S2). To confirm that our YEATS2-YEATS protein was active, we examined its binding to K27-crotonylated H3 (1–34) peptide, to which YEATS2-YEATS reportedly binds stronger (K_D_ = 31.7 μM) than to the K27ac-containing H3 peptide (50). In our ITC analysis, YEATS2-YEATS bound to the K27cr-containing H3 (15–39) peptide with a K_D_ of 340 μM (Fig. S2), indicating that it was active. We also confirmed that the fusion of AG to YEATS2-YEATS had little effect on this binding (Fig. S2). Together, these results suggest that the active YEATS2-YEATS domain used in this study does not significantly prefer any acetylated NCPs over the unmodified NCP.

The GAS41-YEATS domain significantly bound to H3K4-, K9-, K14- and K27-acetylated NCPs (*p* < 0.05; Fig. 2*B*); the binding was strongest to H3K27ac. Statistical significance of YEATS domain binding to Kac is summarized in Figure 2*C*. We also found that the YEATS domains of AF9, ENL, and GAS41 bound significantly to H4K5/K8/K12/K16-tetra-acetylated NCP (Fig. 2, *B* and *C*).

Next, we compared the Kac-binding preferences of YEATS domains to acetylated NCPs and to acetylated histone tail peptides. Binding results obtained using a MODified Histone Peptide Array are shown in Fig. S3. YEATS domains of AF9 and GAS41 reproducibly bound to the histone H3 tail peptide containing K27ac. YEATS2-YEATS bound weakly and nonreproducibly to H3K27ac. These results were consistent with recent reports (21, 31, 32). The YEATS domains of AF9, ENL, and GAS41 bound to the H4 (1–19) tail peptide when it was diacetylated at K5/K8 or tetra-acetylated at K5/K8/K12/K16. The YEATS domains of all four proteins scarcely bound to any of the unmodified histone tail peptides (*i.e.*, H2A, H2B, H3, or H4; Fig. S3).

### The AF9-YEATS domain binds to the unmodified NCP

**Figure 3:**
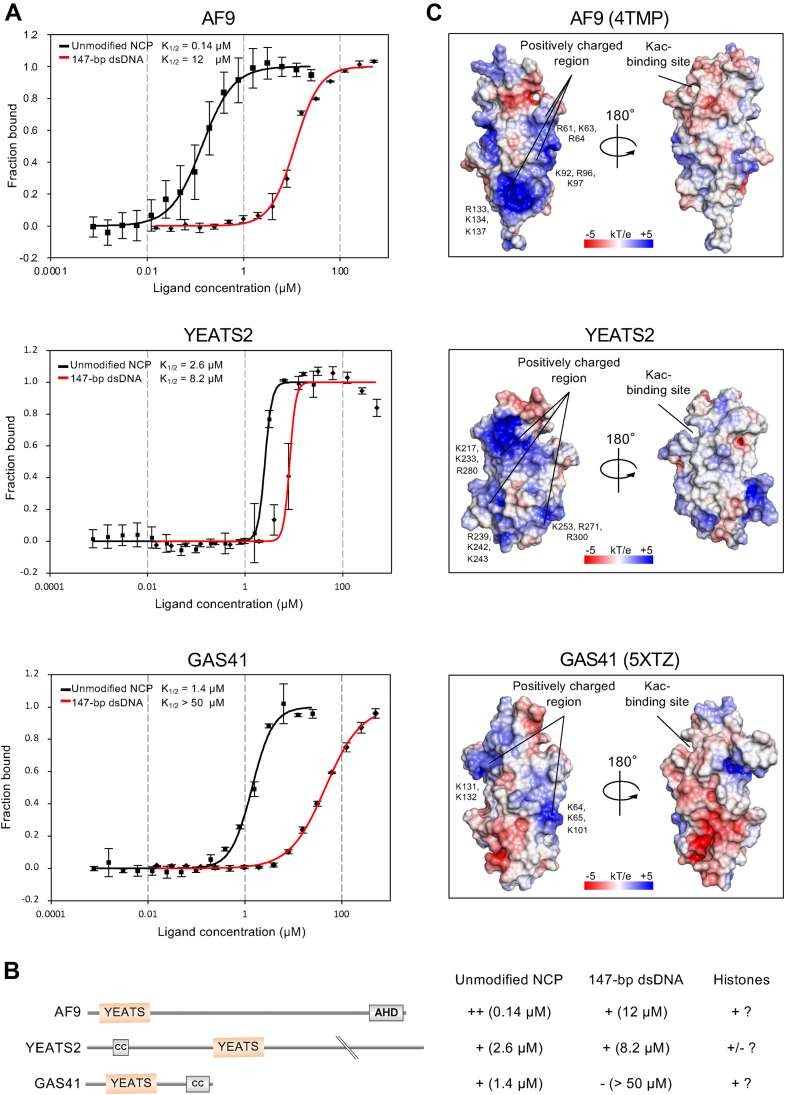
**Binding affinities and crystal structures of AF9-YEATS, YEATS2-YEATS, and GAS41-YEATS.***A*, microscale thermophoresis curves. Means ± SD (n = 3). Fluorescence intensity is normalized to fraction bound (0 = unbound, 1 = bound). *B*, summary of binding activities of the YEATS domains. Relative affinity is shown by ++, +, +/-, and – (no binding), with K_1/2_ values in parentheses. Possible binding to histones is shown. *C*, electrostatic surface potentials (−5 to 5 kT e^−1^) of the YEATS domains of AF9 (PDB ID: 4TMP), YEATS2, and GAS41 (PDB ID: 5XTZ). YEATS, Yaf9, ENL, AF9, Taf14, and Sas5.

We measured the K_1/2_ (the half-saturation concentration for NCP binding) of the interaction between AF9-YEATS and the unmodified NCP or 147-bp dsDNA using microscale thermophoresis. The K_1/2_ value of AF9-YEATS binding to the unmodified NCP composed of 147-bp dsDNA and the histone octamer (0.14 ± 0.01 μM) was 86 times lower (*i.e.*, the binding was stronger) than that for nucleosome-free 147-bp dsDNA (12 ± 0.1 μM; Fig. 3*A*).

In contrast to AF9-YEATS, YEATS2-YEATS, and GAS41-YEATS did not bind to the unmodified NCP in our nucleosome array assay (Fig. 2*B*). The K_1/2_ values for YEATS2-YEATS binding to the unmodified NCP (2.6 ± 0.1 μM) and to nucleosome-free 147-bp dsDNA (8.2 ± 0.6 μM; Fig. 3*A*) indicated that the interaction between YEATS2-YEATS and the unmodified NCP is detectable by microscale thermophoresis. The respective K_1/2_ values for GAS41-YEATS (1.4 ± 0.1 and >50 μM; Fig. 3*A*) also indicated that the interaction between GAS41-YEATS and the unmodified NCP is detectable by microscale thermophoresis. Thus, the binding affinity of YEATS2-YEATS to nucleosome-free dsDNA was similar to that of AF9, whereas that of GAS41-YEATS was much weaker. The results, together with the binding results obtained using the MODified Histone Peptide Array, are summarized in Figure 3*B*.

### Surface potentials of the YEATS domains

The ability of YEATS domains to bind the NCP and nucleosome-free dsDNA is assumed to be regulated by their electrostatic surface potentials (38). The surface potential of AF9-YEATS (PDB ID: 4TMP) is shown in Figure 3*C*. Three major positively charged surface regions in AF9-YEATS are composed of 1) R61, K63, and R64; 2) K92, R96, and K97; and 3) R133, K134, and K137.

To investigate the folding and electrostatic potential of YEATS2-YEATS, we determined the crystal structure of the YEATS2-YEATS apo-form at 1.67 Å resolution. The overall structure was similar to that of AF9-YEATS with an alpha carbon RMSD value of 0.76 Å, indicating the absence of misfolding in our YEATS2-YEATS protein. The electrostatic surface potential of YEATS2-YEATS is shown in Figure 3*C*. Similar to AF9-YEATS, YEATS2-YEATS had three major positively charged surface regions: 1) K217, K233, and R280; 2) K239, K242, and K243; and 3) K253, R271, and R300.

The surface of GAS41-YEATS (PDB ID: 5XTZ) was more negatively charged than those of AF9-YEATS or YEATS2-YEATS (Fig. 3*C*), which was consistent with dsDNA binding to AF9-YEATS and YEATS2-YEATS, but not to GAS41-YEATS.

### The AF9-YEATS domain binds to the H4-multiacetylated NCP

**Figure 4:**
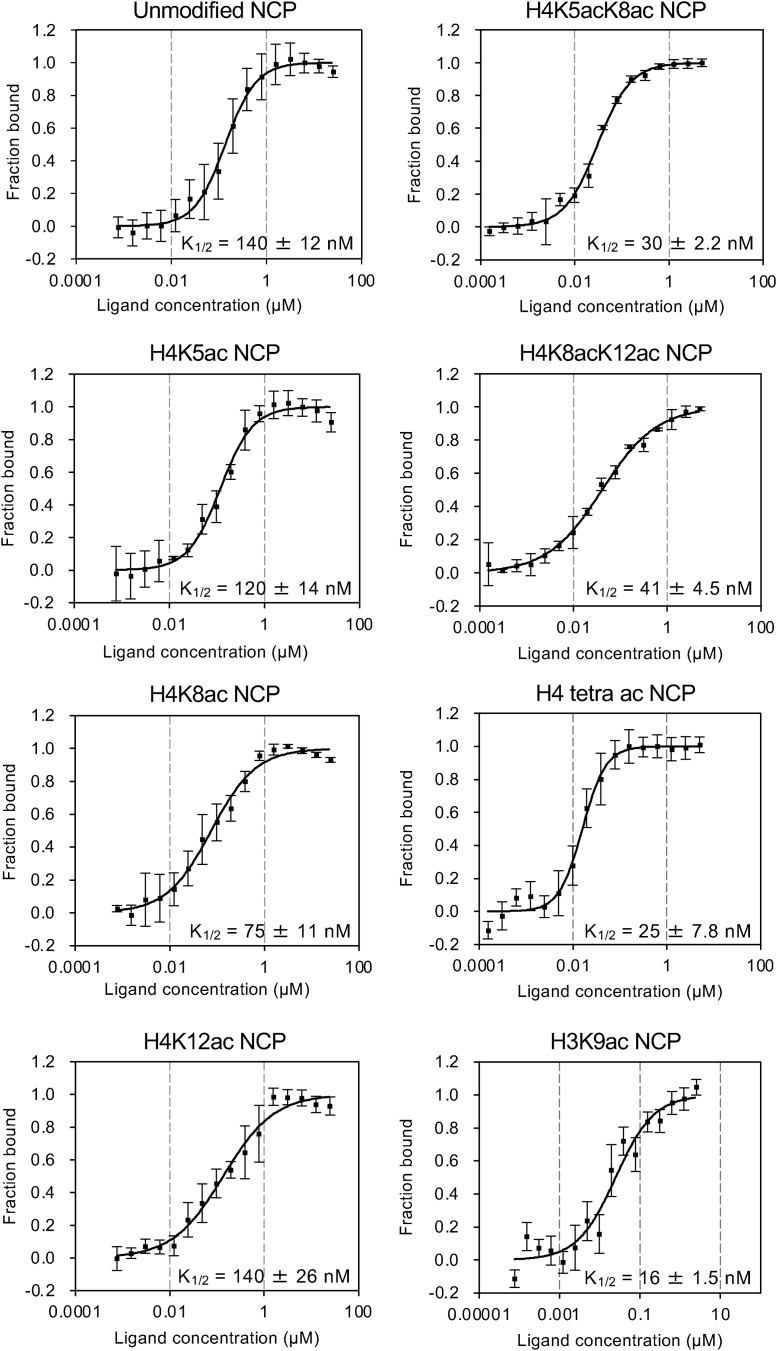
**Microscale thermophoresis curves of AF9-YEATS binding to NCPs.** Fluorescence intensity is normalized to fraction bound (0 = unbound, 1 = bound). NCP, nucleosome core particle; YEATS, Yaf9, ENL, AF9, Taf14, and Sas5.

Using microscale thermophoresis, we measured the affinity of AF9-YEATS binding to the NCP containing either mono-, di-, or tetra-Kac in the N-terminal tail of histone H3 or H4 (Fig. 4). As in the nucleosome array assay (Fig. 2, *B* and *C*), AF9-YEATS most strongly bound to the NCP containing H3K9ac. The K_1/2_ values were 16 ± 1.5 nM for H3K9ac, 120 ± 14 nM for H4K5ac, 75 ± 11 nM for H4K8ac, and 140 ± 26 nM for H4K12ac (Fig. 4). Thus, AF9-YEATS preferentially bound H4K8ac over H4K5ac and H4K12ac, with a K_1/2_ 1.9 times lower than that for the unmodified NCP. AF9-YEATS bound stronger to diacetylated (K_1/2_: 30 ± 2.2 nM for H4K5ac/K8ac; 41 ± 4.5 nM for H4K8ac/K12ac) or tetra-acetylated NCPs (25 ± 7.8 nM for H4K5ac/K8ac/K12ac/K16ac) than to monoacetylated NCPs (Fig. 4). Thus, diacetylation increases AF9-YEATS binding 3.4 times for H4K5ac/K8ac and 4.7 times for H4K8ac/K12ac over the unmodified NCP, and tetra-acetylation at K5/K8/K12/K16 increases it 5.6 times. Overall, the binding affinity of AF9-YEATS to H4-acetylated NCP was strongest for tetra-acetylation, followed by two diacetylations, and was relatively weak for monoacetylation and for the absence of modification.

### Crystal structures of the AF9-YEATS domain complexed with diacetylated H4 peptides

**Figure 5:**
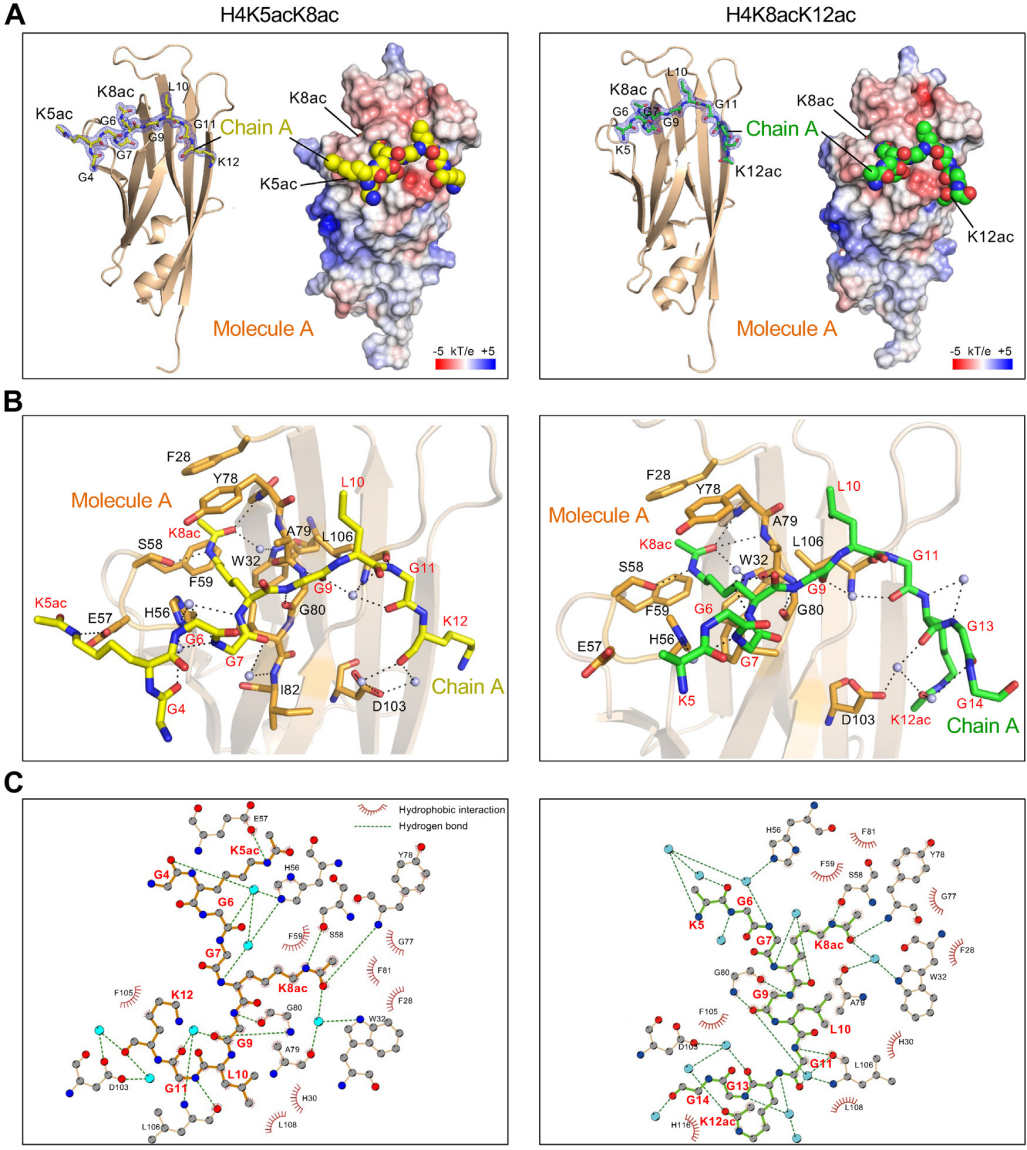
**Crystal structures of the AF9-YEATS domain in complex with H4-diacetylated tail peptides.***A*, the overall structures and electrostatic potential surfaces of the H4 (1–12) K5ac/K8ac complex (*left*) and the H4 (4–16) K8ac/K12ac complex (*right*); atoms in the peptides are depicted as space-filling spheres. *B*, hydrogen-bonding networks between AF9 and the H4K5ac/K8ac peptide (*left*) and the H4K8ac/K12ac peptide (*right*); hydrogen bonds are shown as *black dashes*, AF9 residues are depicted as *orange sticks* and are labeled in *black*, and the peptides are shown as *yellow* (H4K5ac/K8ac) or *green* (H4K8ac/K12ac) sticks and are labeled in *red*. *C*, two-dimensional plots of residues interacting with the H4K5ac/K8ac peptide (depicted in *yellow*) (*left*) and the H4K8ac/K12ac peptide (depicted in *green*) (*right*). *Gray* ball, carbon; *blue* ball, nitrogen; *red* ball, oxygen; *cyan* ball, a water molecule. YEATS, Yaf9, ENL, AF9, Taf14, and Sas5.

In our binding assays based on the nucleosome array or peptide array, the YEATS domains, except YEATS2-YEATS, preferred K5/K8/K12/K16-tetra-acetylated H4 (Fig. 2, *B* and *C*, and S3). The YEATS domain-containing protein Taf14, an ortholog of AF9 in the budding yeast, and GAS41 also bind to multiacetylated histone H3 (37, 51). GAS41 dimerizes through a coiled-coil region in its C-terminal part, and the dimer binds to diacetylated H3 by recognizing a single Kac *via* a single YEATS domain. However, AF9 is monomeric in solution (21), and it is unclear how its YEATS domain recognizes multiacetylated histones, including tetra-acetylated H4. To understand the structural mechanism of this recognition, we tried to solve the crystal structure of the AF9-YEATS domain in complex with the H4 (1–12) K5ac/K8ac, H4 (4–16) K8ac/K12ac, H4 (8–20) K12ac/K16ac, or H4 (1–20) K5ac/K8ac/K12ac/K16ac peptide. Unfortunately, we obtained microcrystals with the latter two peptides with a resolution of >10 Å and thus could not solve their crystal structures. We determined the crystal structures of AF9-YEATS complexed with the K5/K8- (Fig. 5*A*, 1.95 Å resolution) and K8/K12- (Fig. 5*A*, 2.00 Å resolution) diacetylated H4 peptides (Table 1). The crystal forms of AF9-YEATS in complex with K5/K8- and K8/K12-diacetylated peptides both belonged to the space group *P*1, and each asymmetric unit contained two AF9-YEATS molecules (molecules A and B; Fig. S4, *A* and *B*).

**Table 1:** Data collection and refinement statistics

Protein	AF9		YEATS2
Ligand	H4K5ac/K8ac	H4K8ac/K12ac	
Data collection and processing			
X-ray source	BL26B2, SPring-8	BL26B2, SPring-8	BL26B2, SPring-8
Space group	*P*1	*P*1	*C*2
Cell dimensions			
*a*, *b*, *c* (Å)	30.37, 51.70, 56.27	42.96, 44.91, 58.80	90.45, 50.84, 82.63
*α*, *β*, *γ* (°)	89.30, 74.81, 89.87	84.86, 86.09, 66.33	90.00, 100.22, 90.00
Wavelength (Å)	1.000	1.000	1.000
Resolution range (Å)^a^	50.0–1.95 (1.98–1.95)	50.0–2.00 (2.03–2.00)	44.5–1.67 (1.70–1.67)
No. of observed reflections	41,328	51,180	163,041
No. of unique reflections	22,633	26,432	43,069
Redundancy	1.9 (1.8)	2.0 (1.9)	3.8 (3.7)
Completeness (%)	94.1 (80.1)	97.3 (95.8)	100.0 (100.0)
*R*_sym_ (%)^b^	3.8 (20.5)	4.0 (31.6)	4.4 (74.6)
*I*/σ(*I*)	13.9 (3.1)	13.8 (1.9)	11.6 (1.6)
Model refinement			
Resolution range (Å)	50.0–1.95 (1.98–1.95)	50–2.00 (2.03–2.00)	44.5–1.67 (1.70–1.67)
*R*_work_/*R*_free_ (%)^c^	17.68/20.48	20.25/22.8	17.37/20.19
RMSD for bond length (Å)	0.005	0.006	0.003
RMSD for bond angles (°)	1.137	1.115	1.140
No. of nonhydrogen atoms			
Protein	2333	2426	2104
Water molecules	141	271	230
Mean *B*-factors (Å^2^)			
Protein	36.13	31.30	38.08
Water molecule	41.92	45.96	49.17
Residues in the Ramachandran plot			
Most favored (%)	98.9	99.3	98.0
Allowed (%)	1.1	0.7	2.0
Outliers (%)	0	0	0
PDB entry	7EIC	7EID	7EIE

a Values in parentheses are for the outermost resolution shell.

b *R*_sym_ = (∑_*h*_∑_*i*_|*I*_*hi*_ –‹*I*_*h*_›|/∑_*h*_∑_*i*_|*I*_*hi*_|), where *h* indicates unique reflection indices and *i* indicates symmetry equivalent indices.

c *R*_work_ = ∑|F_obs_–F_calc_|/∑F_obs_ for all reflections; *R*_free_ was calculated by using randomly selected reflections.

Molecule A in complex with the H4K5ac/K8ac peptide (chain A) and molecules A and B in complex with two H4K8ac/K12ac peptides (chains A and B) recognized H4K8ac at the aromatic cage (Figs. 5*B*, S4, *A* and *B*). Molecule B in the H4K5ac/K8ac peptide complex did not bind the peptide at the aromatic cage because the aromatic cage was closed by conformational changes around Y78 in AF9 (Fig. S4*C*). The positively charged R61 and R64 residues of molecule B hydrogen bonded with chain A in both complexes (Fig. S4, *A* and *B*). The electron density of H4K5ac was detected in the H4K5ac/K8ac peptide complex structure, but the position of the side chain of unmodified H4K5 in the H4K8ac/K12ac peptide complex could not be determined because of low electron density (Fig. 5*A*). K5ac and K12ac were located outside the aromatic cage; Nε of H4K5ac hydrogen bonded with E57 (Fig. 5*B*, left) and the added acetyl group of H4K12ac hydrogen bonded with D103 *via* a water molecule (Fig. 5*B*, right). Outside this cage, the main chain of unmodified H4K12 in the H4K5ac/K8ac peptide complex also hydrogen bonded with D103 *via* two water molecules, whereas its side chain was oriented toward the solvent (Fig. 5*B*, left). Two-dimensional plots of residues interacting with the H4K5ac/K8ac or H4K8ac/K12ac peptide are shown in Figure 5*C*. The added acetyl group of H4K12ac interacted hydrophobically with H116 of AF9. These structures indicate that the aromatic cage of AF9-YEATS recognizes a single Kac (H4K8ac) even when the nearby lysines in the H4 N-terminal tail are acetylated.

The interaction modes of AF9-YEATS with H4K8ac and with H3K9ac (21) were the same: the aromatic cage of AF9-YEATS hydrophobically interacted with the aromatic residues F28, H56, F59, Y78, and F81; and the amide and the carbonyl oxygen of the Kac side chain formed hydrogen bonds with the hydroxyl group of S58 and the backbone amide of Y78 (Fig. 5*B*).

### Binding of the AF9-YEATS domain to acetyllysine outside the aromatic cage

**Figure 6:**
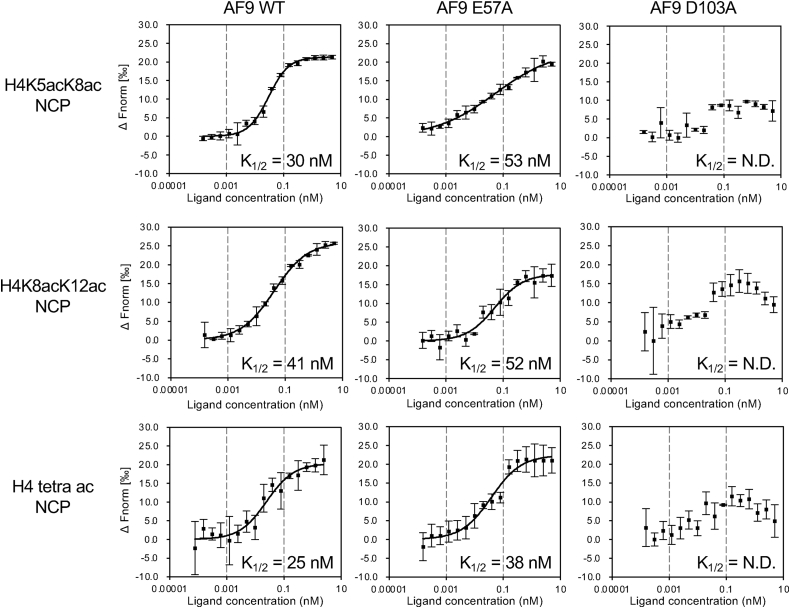
**Microscale thermophoresis curves of AF9-YEATS mutant binding to multi-acetylated NCPs.** Measured fluorescence values are shown as normalized fluorescence (Fnorm), defined as F1/F0, where F0 and F1 are the fluorescence values prior to and after infrared laser activation, respectively. ΔFnorm (‰) is 10 × (Fnorm_bound_ − Fnorm_unbound_). NCP, nucleosome core particle; YEATS, Yaf9, ENL, AF9, Taf14, and Sas5.

Finally, we investigated whether the AF9-YEATS residues that interacted with the Kac adjacent to K8ac of the H4 peptide in the crystal are important for the interaction in solution. To this end, we replaced H4K5ac-interacting E57 or H4K12ac-interacting D103 of AF9-YEATS with alanine and used the mutated proteins and multiacetylated NCPs in microscale thermophoresis (Fig. 6). The interaction was weaker for the E57A mutant than for the WT with any of the multiacetylated NCPs tested. The increase in K_1/2_ toward K5/K8-diacetylated and tetra-acetylated NCPs was 1.8- and 1.5-fold, respectively. On the other hand, the increase of K_1/2_ toward K8/K12-diacetylated NCPs was 1.3-fold, which is the lowest among the three multiacetylated NCPs. This result suggests that the E57 residue is important for the interaction with multiacetylated NCPs containing H4K5ac. The D103A mutant did not interact with any of the multiacetylated NCPs, suggesting that this mutation affected protein folding.

### Binding of multiacetylated H3 peptides by the AF9-YEATS domain

Intriguingly, Taf14 has higher affinity for multiacetylated histone H3 than for the monoacetylated one (51). To investigate whether AF9-YEATS preferentially binds to multiacetylated H3 and H4, we used ITC to measure its binding affinity toward five monoacetylated or diacetylated H3 (1–19) peptides (Fig. S5). Among peptides monoacetylated at K4, K9, or K14, AF9-YEATS specifically bound to the K9-acetylated H3 peptide with a K_D_ of 7.0 μM. Additional acetylation at K4 slightly increased its affinity (K_D_ = 5.8 μM), whereas that at K14 slightly decreased it (K_D_ = 8.5 μM). These results suggest that, similar to the role of K5ac in the K8ac-mediated interaction of the H4 tail peptide, K4ac in the H3 tail peptide contributes to the K9ac-mediated interaction outside of the YEATS domain cavity.

## Discussion

Identification of the preferences of chromatin-associated factors for PTMs is important in understanding their action and mechanism. In a few available reports on the development of PTM-containing nucleosome libraries, these libraries were prepared by native chemical ligation, that is, by conjugating a PTM-containing histone tail peptide with the histone core regions (33, 52). Through genetic code reprogramming and biochemical validation using HDAC6 (Fig. 1) and bromodomains (Fig. S1), we established an NCP-based assay platform and used it to compare the Kac-binding preferences of YEATS domains at the histone peptide and nucleosome levels (Fig. 2). Theoretically, the methodology developed here is applicable not only to histones and the NCP but to any PTM-containing proteins of interest. Because histones are composed of an unfolded N-terminal tail region and a folded C-terminal core region, they are ideal models for the native chemical ligation method. Our methodology may be advantageous over native chemical ligation when applied to the position-specific introduction of Kac into a folded region of a protein other than a histone. Several pyrrolysyl-tRNA synthetase mutants introduce crotonylated lysine site specifically (53, 54), so it is theoretically possible to prepare a nucleosome containing a site specifically crotonylated histone. The crotonylated histone is reportedly recognized by the YEATS domain and regulates transcription (24). Using the NCP-based assay, it would be possible to apply it to a variety of epigenetic reader proteins to investigate their binding properties to crotonylated nucleosomes that were not revealed by the histone peptide-based assay.

Because the histone peptides in the peptide array are unfolded, the YEATS domain can access all the acetylated residues in histone peptides without any steric hindrance, whereas histone tails protrude from the structured NCP and the access of the YEATS domain to the acetylated histone tails may be sterically hindered by the NCP. Thus, the NCP-based binding assay better reflects the chromatin environment in the nucleus than the peptide-based binding assay does; the former assay excludes artifactual binding. Although YEATS2-YEATS reportedly binds a K27ac-containing H3 peptide (31), our YEATS2-YEATS protein scarcely bound to an NCP containing H3K27ac and did not significantly prefer it over the unmodified NCP (Fig. 2, *B* and *C*). In our ITC assay, YEATS2-YEATS did not bind to the K27-acetylated H3 peptide but bound to the K27-crotonylated H3 peptide (Fig. S2). Peptide array assay showed that YEATS2-YEATS weakly and nonreproducibly bound to the K27-acetylated H3 peptide (Fig. S3). Hence, although we cannot exclude the interaction between YEATS2-YEATS and H3K27ac at the peptide level, our NCP-based binding assay revealed that YEATS2-YEATS did not prefer any of the examined H3/H4 acetylations.

This assay is also useful for detecting and evaluating binders that interact with DNA or histones but not with the PTM. In particular, this study suggests that different YEATS domains interact with different sets of components (dsDNA, histones, or both) of the unmodified NCP (Fig. 3*B*). Using fluorescence spectroscopy assay, Klein *et al*. (38) have found that AF9-YEATS may bind to the unmodified NCP owing to its DNA-binding ability: it binds to 15-bp dsDNA (K_D_ = 47 μM) and to 20-bp dsDNA (K_D_ = 57 μM). In our microscale thermophoresis assay (Fig. 3, *A* and *B*), AF9-YEATS bound to 147-bp dsDNA and 86 times stronger to the unmodified NCP (Fig. 3*A*). These results suggest that AF9-YEATS interacts with the unmodified NCP through DNA and also substantially through histones (Fig. 3*B*).

YEATS2-YEATS bound much weaker to the unmodified NCP than AF9-YEATS did, but it bound to 147-bp dsDNA slightly stronger than AF9-YEATS did. On the electrostatic potential maps, AF9-YEATS had no apparent positively charged surface region near the Kac-binding site, whereas YEATS2-YEATS had several positively charged residues, such as K217, K233, and R280 (Fig. 3*C*); these positively charged residues may interact with dsDNA and thus enhance the dsDNA-binding ability of YEATS2-YEATS. The affinity ratio of YEATS2-YEATS toward the unmodified NCP *versus* 147-bp dsDNA was small (3.2). Given these binding properties, YEATS2-YEATS may interact quite weakly with histones (Fig. 3*B*).

GAS41-YEATS may interact with the unmodified NCP through histones because it had considerable affinity to the unmodified NCP whereas it bound weakly to 147-bp dsDNA (Fig. 3*B*). Overall, the binding affinity of YEATS domains for the unmodified NCP cannot be simply explained by their interaction with DNA, and interaction with the histones within the NCP presumably matters (Fig. 3*B*). We attempted to identify the residue(s) of AF9-YEATS that interact(s) with the unmodified NCPs by NMR measurements using the transferred cross-saturation technique, cocrystal structure analysis, and cryo-EM. However, these experiments did not uncover the mechanism of AF9-YEATS binding to the unmodified NCP because of problems with sample preparation.

We solved the crystal structures of AF9-YEATS complexed with the K5/K8- and K8/K12-diacetylated H4 peptides (Fig. 5*A*). In these asymmetric units, two AF9-YEATS molecules seemingly interacted with each other *via* positively charged residues, such as R61 and R64, and the H4 peptide (Fig. S4, *A* and *B*). However, AF9 is monomeric in solution (21), and these positively charged residues are responsible for DNA binding (38). Because the AF9-YEATS domain cannot dimerize in solution and the interaction between the asymmetric AF9-YEATS molecule and the H4 peptide competes with the reported DNA binding, the H4 peptide-binding mode of the AF9-YEATS dimer observed in the crystal is probably caused by crystal packing and may not occur in solution. Thus, the increased affinity to multiple Kac is not due to the dimerization of AF9-YEATS and is presumably attributable to the recognition between a single aromatic cage of AF9-YEATS and a single Kac, accompanied by additional recognition of another nearby Kac within the same histone tail by the residues (*i.e*., E57 and D103) located outside the aromatic cage.

Intriguingly, when another Kac was present near K8ac in the histone H4 tail (*e.g*., K5ac), it interacted with AF9-YEATS (Fig. 4) at the surface outside of the aromatic cage of the YEATS domain in the crystal (Fig. 5, *B* and *C*). Our biochemical analysis indicates that, at least *via* E57, AF9-YEATS senses K5ac adjacent to K8ac in the H4 tail, which additively increases the affinity toward the NCP (Fig. 6). Similarly, AF9-YEATS may also sense K4ac adjacent to K9ac in the H3 tail (Fig. S5). Hence, the simultaneous presence of two or more Kac in the H3 and/or H4 tail may increase the number of such interactions using both the YEATS domain cavity and its outer surface.

Another Kac-binding domain, the N-terminal bromodomain of the members of the bromodomain and extra-terminal domain family, can bind to the K5/K8-diacetylated histone H4 tail through simultaneous recognition of two Kac in the same bromodomain cavity (Fig. S4*D*) (30, 55). Intriguingly, the mode of K5/K8 diacetylated H4 recognition by the AF9-YEATS domain was completely different from that by the bromodomain of the bromodomain and extra-terminal domain family. The AF9-YEATS domain primarily recognized H4K8ac regardless of acetylation at K5 or K12. Importantly, newly synthesized H4 is diacetylated in the cytoplasm at K5 and K12, and other lysine residues in the N-terminal tail, including K8, can be further acetylated in the nucleus (56, 57). Therefore, the mode of primary binding to K8ac and secondary binding to K5ac or K12ac by the AF9-YEATS domain provides a unique molecular basis for sensing the hyperacetylation of H4 in the nucleus. Approaches such as coimmunoprecipitation and ChIP-seq are needed to establish whether the direct binding of AF9 to the H4 multiacetylated NCP is involved in the regulation of a nuclear function such as gene expression, as in the case of AF9 binding to H3K9ac (21).

In conclusion, we established a binding assay based on site specifically acetylated NCPs and found that the AF9-YEATS domain binds to H4-multiacetylated NCPs. The crystal structures of the AF9-YEATS domain in complex with H4 peptides revealed that K8ac is recognized within the aromatic cage of the YEATS domain, whereas the nearby K5ac and K12ac contribute to the binding outside of the aromatic cage. These structural and mutagenesis analyses provide a binding model in which the Kac-binding affinity of AF9-YEATS increases additively with the number of Kac in the histone tail. Our analyses suggest that the YEATS domain of AF9 binds the NCP mainly through DNA and histones, that of YEATS2 binds mainly through DNA, and that of GAS41 binds mainly through histones. The methodology presented here will enable preparation of a library of nucleosomal beads-on-a-string. Characterization of chromatin-associated factors in the context of higher-order chromatin with PTMs will aid in understanding their functions in the natural chromatin environment in the nucleus.

## Experimental procedures

### Preparation of histones and biotinylated H2B

Unmodified human histones (H2A, H2B, H3.1, and H4) were expressed and purified as previously described (13). All histones were lyophilized, suspended in 10 mM Tris-HCl buffer (pH 7.5) containing 1 mM EDTA. To prepare biotinylated H2B, an avidin-tag and TEV protease recognition site-containing sequence were inserted at the C-terminus of H2B; the fusion protein was purified as avidin-tagged H2B. Biotinylation was performed at 30 °C for 16 h by incubating 40 μM avidin-tagged H2B and 0.8 μM recombinant biotin ligase A in 10 mM bicine buffer (pH 8.3) containing 100 μM D-biotin, 3 mM ATP, and 10 mM MgCl_2_. Biotinylation was confirmed by Western blotting using Avidin peroxidase conjugate (Abcam, ab59653).

### Preparation of specifically acetylated histones H3 and H4

The Kac-containing human histones (K4-, K9-, K14-, K27-, and K36-acetylated H3.1; K5-, K8-, K12-, K16-, K5/K8-, K5/K12-, K8/K12-, K12/K16-, and K5/K8/K12/K16-acetylated H4) were prepared as previously described (13, 43, 58). Human H3.1 or H4 complementary DNA (cDNA) with the codon(s) for the specified residue(s) replaced with the TAG triplet(s) and a terminal TAA stop codon was prepared for protein synthesis in the coupled transcription–translation cell-free system (13). In this system, mRNAs with UAG triplet(s) at the specified position(s) are transcribed with T7 RNA polymerase and UAG is efficiently recognized by tRNA^Pyl^ acylated with Kac, yielding a protein with Kac at specific positions in milligram quantities. Synthesized histones were purified as described (13).

### Western blotting

Purified histones were electrophoresed in a 17.5% SDS polyacrylamide gel and transferred onto the polyvinylidene difluoride membrane at 15 V for 1 h by the semidry method. The membrane was blocked with TBS-T buffer containing 5% nonfat dried milk at room temperature for 1 h and then washed with TBS-T (twice for 10 min each). Membranes were incubated at 4 °C for 12 h with antibodies (from Millipore unless indicated otherwise) against H3 C-terminal region (07-690), H3K4ac (ABE223), H3K9ac (07-352), H3K14ac (Abcam, ab52946), H3K27ac (07-360), H3K36ac (07-540), H4 C-terminal region (Abcam, ab10158), H4K5ac (07-327), H4K8ac (07-329), H4K12ac (07-595), or H4K16ac (07-328). The membranes were then washed with TBS-T (4 times for 5 min each) and incubated with peroxidase-conjugated anti-mouse IgG (GE Healthcare, NA931) or anti-rabbit IgG (GE Healthcare, NA934) at room temperature for 1 h. The membranes were then washed with TBS-T (4 times for 5 min each) and were detected using enhanced chemiluminescence (Chemi-Lumi One Super: 02230-30).

### Reconstitution of H2B-biotinylated NCPs

The H3.1- or H4-acetylated NCPs along with the unmodified NCPs were reconstituted as described (13, 59), using Kac-containing H3.1 or H4, unmodified histones, biotinylated H2B, and 147-bp palindromic human α-satellite DNA. Briefly, purified histones were mixed at an equimolar ratio in a solution containing 7 M guanidine-hydrochloride and 10 mM DTT and dialyzed against 10 mM Tris-HCl buffer (pH 7.5) containing 2 M KCl and 5 mM DTT at 4 °C for 4 h; dialysis buffer was exchanged 4 times. Histone octamers were concentrated in Amicon Ultra-15 centrifugal units (Merck Millipore, MWCO 30 kDa) and purified by gel filtration chromatography using a HiLoad 16/60 Superdex 200 column (GE Healthcare). Histone octamers and 147-bp DNA were mixed at a 1.0 : 1.1 M ratio in 10 mM Tris-HCl buffer (pH 7.5) containing 2 M KCl, 1 mM EDTA, and 1 mM DTT. The solution was placed in a dialysis membrane bag (Spectrum, MWCO 6-8 kDa, cat. no. 132653) and dialyzed against the same buffer at 4 °C for 4 h. The concentration of KCl was then gradually decreased by diluting for 30 h with 10 mM Tris-HCl buffer (pH 7.5) containing 1 mM EDTA and 1 mM DTT using a peristaltic pump. The reconstituted NCP samples were incubated at 55 °C for 2 h. To separate free DNA, MgCl_2_ was added to the reconstituted NCP samples at a final concentration of 12 mM. The NCPs were precipitated by centrifugation at 17,500*g* for 10 min at 4 °C and suspended in 10 mM Tris-HCl buffer (pH 7.5) containing 1 mM EDTA and 1 mM DTT.

### Nucleosome deacetylation and deacetylation inhibition assays

The NCPs for deacetylation assays were reconstituted using unmodified H2B. In each deacetylation reaction, 200 ng of the Kac-containing NCP was incubated with 50, 100, 200, or 400 ng of HDAC6 (SignalChem; cat. no. H88-30 G) in 20 μl at 37 °C for 16 h. In the inhibitor assays, TSA was added to the reaction mixture not containing NCP to a final concentration of 1 μM and preincubated at 37 °C for 1 h; NCP monoacetylated either at K5, K8, K12, or K16 of H4 was then added. After the reactions, samples were spotted on a nitrocellulose membrane (BioTrace NT Nitrocellulose Transfer Membrane, 0.2 μm, Pall Corporation, cat. no. 66485) and immunoblotted using H4-specific antibodies, each recognizing acetylation at K5, K8, K12, or K16.

### Preparation of bromodomains and YEATS domains

cDNAs-encoding bromodomains or YEATS domains were fused with a histidine-tagged sequence containing a SUMO protease digestion site at their N-termini and a full-length AG cDNA (47) at their C-termini. The following fragments of human bromodomain proteins were used: BAZ2B (residues 2054–2168), BRD2-BD1 (74–194), BRD2-BD2 (348–455), BRD2-BD1+BD2 (74–455), BRD3-BD1 (33–153), BRD3-BD2 (306–421), BRD3-BD1+BD2 (33–421), BRD4-BD1 (55–168), BRD4-BD2 (346–459), BRWD3-BD2 (1295–1384), CBP (1082–1197), p300 (1038–1161), PB1-BD2 (175–291), PCAF (715–831), SMARCA2 (1367–1507), SMARCA4 (1448–1569), TAF1L-BD1 (1404–1502), TAF1L-BD1+BD2 (1378–1657), TRIM28 (624–812), and WDR9-BD2 (1310–1430). Regarding BRDT, mouse BRDT-BD1+BD2 (26–375) was used. The following fragments of YEATS domain proteins were used: AF9 (residues 1–138), ENL (1–138), YEATS2 (200–349), and GAS41 (15–159). A T7 promoter sequence was inserted at the 5′ ends of the AG-fused protein expression constructs, and the cDNA cassettes were inserted into a pCR2.1 TOPO plasmid by using a TA cloning kit (ThermoFisher Scientific, cat. no. 450641). The AG-fused proteins were produced in cell-free protein synthesis reactions in 6 ml with 2 μg/ml template DNA, essentially as previously described (60). Then, the reaction mixtures were centrifuged at 28,000*g* for 20 min at 4 °C and the AG-fused proteins were purified from the supernatants by HisTrap HP column chromatography (GE Healthcare). The columns were washed with 10 mM Tris-HCl buffer (pH 8.0) containing 1 M NaCl and 20 mM imidazole, and bound proteins were eluted with 10 mM Tris-HCl buffer (pH 8.0) containing 500 mM NaCl and 500 mM imidazole. Eluted fractions were dialyzed against 10 mM Tris-HCl buffer (pH 8.0) containing 150 mM NaCl and 1 mM DTT and used for binding assay.

For the assay using the MODified Histone Peptide Array, the same cDNA sequences encoding human YEATS domains were amplified by PCR and subcloned into a pCR2.1 vector with a GST-encoding sequence inserted after an N-terminal polyhistidine tag. For microscale thermophoresis measurements, a similar construct but without GST was used. Site-directed mutagenesis of AF9-YEATS (residues 1–138) was conducted by PCR using the DpnI restriction enzyme and the following primer sets: 5′-CCT TCA CGC ATC CTT TCC TCG TCC TAA AC-3′ (forward) and 5′-GGA AAG GAT GCG TGA AGG TGA AAC ACC AC-3′ (reverse) for E57A and 5′-CGA CTA TGA CCT GTT TCT GCA TCT CG-3′ (forward) and 5′-GCA GAA ACA GTG CAT AGT CGA AGC GAA C-3′ (reverse) for D103A. The introduced mutations were verified by DNA sequencing. For X-ray structural analysis, the cDNAs encoding AF9-YEATS (residues 1–138) and YEATS2-YEATS (202–338) were amplified by PCR and subcloned into the pCR2.1 vector encoding GST and the polyhistidine tag. For ITC experiments, the cDNAs encoding AF9-YEATS (residues 1–138) and YEATS2-YEATS (200–349) were inserted into the same vector as for X-ray analysis. The expression using this vector has been previously described (61). All YEATS domain proteins were purified on a HisTrap HP column (GE Healthcare), and the eluted fractions were loaded on a HiLoad Superdex 200 16/60 column (GE Healthcare) equilibrated with Tris-HCl buffer (pH 7.6) containing 500 mM NaCl and 1 mM DTT.

### NCP-based binding assay

The surface of a Pierce streptavidin-coated high-capacity 96-well black plate (ThermoFisher Scientific, cat. no. 15503) was washed 3 times with 10 mM Tris-HCl buffer A (pH 7.5) containing 150 mM NaCl and 0.05% Tween-20. In each well, 5 pmol of the unmodified or acetylated NCPs containing biotinylated H2B were immobilized at 4 °C for 16 h, NCP solution was removed, and the wells were washed 3 times with buffer B (10 mM Tris-HCl, pH 7.5, containing 150 mM NaCl). Then, buffer B containing 0.5% nonfat dried milk was added to each well and was incubated at 25 °C for 1 h, and the wells were washed 3 times with buffer B. AG-fused bromodomain or YEATS domain proteins (25 pmol each) were added to each well, incubated at 25 °C for 1 h, and the wells were washed 3 times with buffer B. Fluorescence intensity was measured in an EnVision multilabel plate reader (PerkinElmer) at an excitation wavelength of 485 nm and an emission wavelength of 535 nm. The binding ratio was calculated for each well by dividing fluorescence intensity after the final wash (bound fraction) by the initial fluorescence intensity at the addition of AG-fused protein. All binding experiments were performed in triplicate. A two-tailed Student’s *t* test was performed for statistical analysis of preference for acetylated *versus* unmodified NCPs. We classified *p* value <0.05 as ∗, *p* value <0.01 as ∗∗.

### Peptide array-based binding assay

Assays were performed using the MODified Histone Peptide Array (Active Motif) following the manufacturer’s protocol. Briefly, the arrays were blocked by incubation in 10 mM Tris-HCl (pH 7.5) containing 150 mM NaCl, 0.05% Tween-20, and 5% nonfat dried milk at 4 °C overnight. The arrays were then washed with 50 mM Tris-HCl (pH 7.6) containing 250 mM NaCl, 0.1% NP-40, and 10% glycerol and incubated with the 10 μM YEATS domain at 4 °C overnight with gentle agitation. Arrays were washed with 10 mM Tris-HCl (pH 7.5) containing 150 mM NaCl and 0.05% Tween-20 and incubated with anti-GST primary antibodies at 4 °C overnight (1:2000 dilution) and then with anti-rabbit HRP secondary antibodies (1:2500 dilution). Protein binding was detected by Chemi-Lumi One Super (Nacalai Tesque). Signal intensities were quantified by using the Protein Array Analyzer for ImageJ (written by Gilles Carpentier, 2010. The macro is available online: http://rsb.info.nih.gov/ij/macros/toolsets/Protein Array Analyzer.txt).

### Microscale thermophoresis

Recombinant YEATS domain proteins were labeled by using a His-tag labeling kit (NanoTemper Technologies, cat. no. MO-L018). Buffer for the labeled proteins and NCPs was changed to 10 mM Hepes-NaOH (pH 7.4) containing 150 mM NaCl. Measurements were performed at room temperature on a Monolith NT.115 instrument (NanoTemper Technologies), using the manufacturers’ protocol. Each assay was performed in biological triplicate with 147-bp dsDNA, unmodified NCP, monoacetylated NCP (H3K9ac, H4K5ac, H4K8ac, and H4K12ac), diacetylated NCP (H4K5ac/K8ac and H4K8ac/K12ac), and H4K5/K8/K12/K16-tetra-acetylated NCP. The data were fitted to the Hill equation using the NT analysis software (NanoTemper Technologies).

### Isothermal titration calorimetry

Measurements were performed at 15 °C in 20 mM Tris-HCl (pH 7.6) containing 500 mM NaCl on a MicroCal Auto-iTC200 microcalorimeter (Malvern). Protein (100 μM, ca. 400 μl) was loaded into the sample cell, and acetylated peptides (1 mM) were loaded into the injection syringe. The collected data were analyzed using Origin 7 SR4, ver. 7.0552 software (OriginLab Corporation) supplied with the instrument to yield enthalpies of binding (ΔH) and K_D_. In all cases, a single binding site model was used.

### Crystallization and structural analysis

Crystallization was performed by the sitting-drop vapor-diffusion method at 20 °C by mixing equal volumes of the YEATS domain of AF9 or YEATS2 (each at 7 mg/ml) and a reservoir solution. Crystals of the AF9-YEATS domain complexed with the H4K5ac/K8ac peptide were grown in 100 mM Bis-Tris buffer (pH 6.5) containing 100 mM ammonium acetate and 25% PEG 3350. Crystals of the AF9-YEATS domain complexed with the H4K8ac/K12ac peptide were grown in 200 mM ammonium citrate tribasic buffer (pH 7.0) containing 19% PEG3350. Crystals of the apo-form YEATS2-YEATS domain were grown in 100 mM Tris-HCl buffer (pH 8.5) containing 2 M NaCl. The crystals were briefly soaked in a cryoprotectant drop composed of the reservoir solution supplemented with 20% glycerol and then flash-cooled in liquid nitrogen for X-ray diffraction data collection. The datasets were collected at the beamline BL26B2, SPring-8 (Harima), and were processed with XDS and HKL2000 (62, 63). Crystal structures were determined by molecular replacement using MOLREP with the structure of AF9 (PDB ID: 4TMP) as the search model. Model building was accomplished with Coot (64, 65), and structural refinement was performed with REFMAC and PHENIX (66, 67). Two-dimensional interaction plots were drawn with LIGPLOT (68). The structural models in the figures were drawn using PyMOL software (Schrodinger, LLC). All electrostatic surface maps were calculated using the APBS tool in a range of −5 kT e^−1^ to +5 kT e^−1^ (69).

## Data availability

The structural coordinates have been deposited in the Protein Data Bank under the accession codes 7EIC for the AF9-YEATS•H4 (1–12) K5ac/K8ac complex, 7EID for the AF9-YEATS•H4 (4–16) K8ac/K12ac complex, and 7EIE for the YEATS2-YEATS domain.

## Supporting information

This article contains supporting information.

## Conflict of interest

The authors declare that they have no conflicts of interest with the contents of this article.

## Supporting information

Kikuchi_et_al_Final_Supporting_information
