# Supplementary material for: Elucidation of binding preferences of YEATS domains to site-specific acetylated nucleosome core particles
Source: J Biol Chem. 2022 Jun 19;298(8):102164. doi: 10.1016/j.jbc.2022.102164 (PMC9293779; doi:10.1016/j.jbc.2022.102164)
Supplement: Kikuchi_et_al_Final_Supporting_information [file mmc1.pdf]

Elucidation of binding preferences of YEATS domains to site-specific acetylated nucleosome core particles

Masaki Kikuchi[1†], Satoshi Morita[1†], Mie Goto[1†], Masatoshi Wakamori[1], Kazushige Katsura[1], Kazuharu Hanada[1], Mikako Shirouzu[1] and Takashi Umehara[1,2*]

[1]RIKEN Center for Biosystems Dynamics Research, 1-7-22 Suehiro-cho, Tsurumi, Yokohama 230-0045, Japan

[2]PRESTO, Japan Science and Technology Agency (JST), 4-1-8 Honcho, Kawaguchi, Saitama 332-0012, Japan

[†]Contributed equally to this work

*Corresponding author; e-mail address: takashi.umehara@riken.jp

Supporting Information

Figure S1. Binding preferences of human bromodomain proteins determined using an NCP-binding assay system.

Figure S2. Isothermal titration calorimetry (ITC) analysis of the interaction between the YEATS2-YEATS domain and histone H3 (15–39) peptides.

Figure S3. Preferences of YEATS domains determined using the modified histone peptide array.

Figure S4. Crystal structures of AF9 in complex with di-acetylated H4 peptides in the asymmetric unit.

Figure S5. ITC analysis of the interaction between the AF9-YEATS domain and histone H3 (1–19) peptides.

Supplementary Figure S1

A

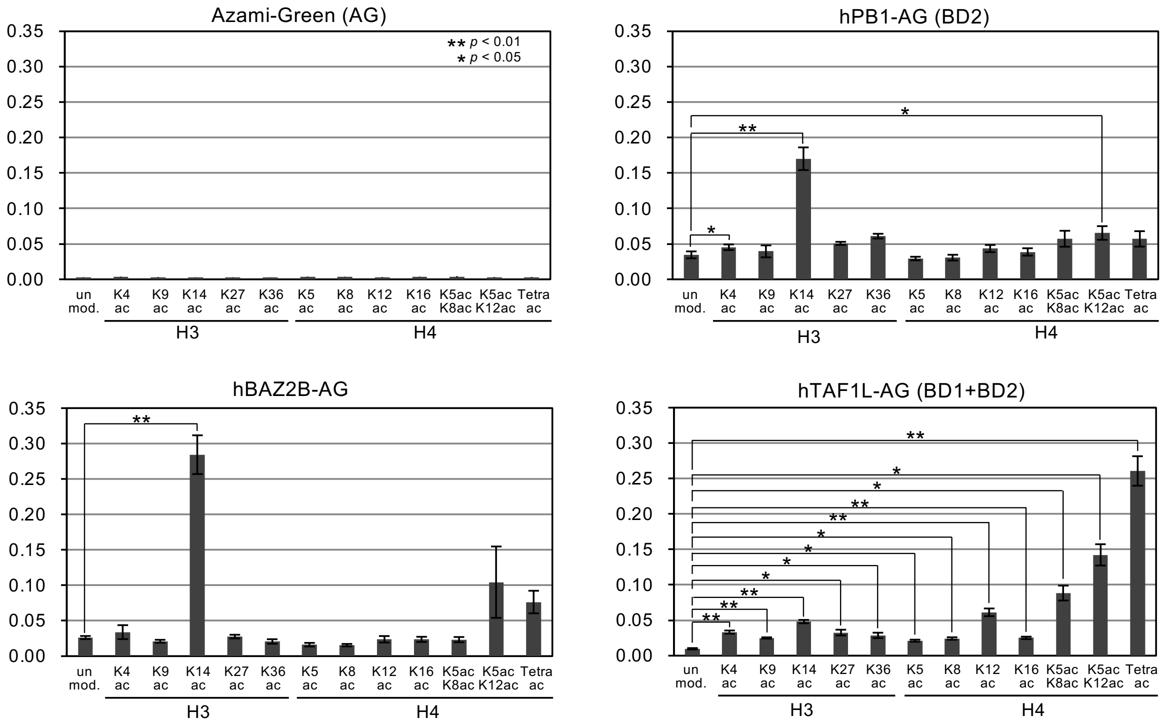

B

	H3					H4						
	K4ac	K9ac	K14ac	K27ac	K36ac	K5ac	K8ac	K12ac	K16ac	K5ac/K8ac	K5ac/K12ac	Tetra ac
BAZ2B			*									
BRD2-BD1		*						*		*	*	**
BRD2-BD2						**	**					
BRD2-BD1+BD2					*							
BRD3-BD1								*		**	*	**
BRD3-BD2	**	*	*		*							*
BRD3-BD1+BD2					*	*						
BRD4-BD1										**	*	**
BRD4-BD2		**	**	*	*							**
BRDT-BD1+BD2	**	*	**	**	*							*
BRWD3-BD2			*									
CBP								**		**	*	**
p300					*			*		**	*	**
PB1-BD2	*		**								*	
PCAF			**					*		**		*
SMARCA2		**										
SMARCA4			*	*								
TAF1L-BD1											**	
TAF1L-BD1+BD2	**	**	**	*	*	*	*	**	**	*	*	**
TRIM28			*									
WDR9-BD2		*	*		*	**	*	**		**	**	**

Figure S1. Binding preferences of human bromodomain proteins determined using an NCP-binding assay system. *A*, the binding ratio of Azami-Green (AG) (top left), AG-fused bromodomain 2 (BD2) of human PB1 (top right), AG-fused BAZ2B bromodomain (bottom left) and AG-fused TAF1 double bromodomain (BD1+BD2) (bottom right). Acetylated residue(s) and histone subunit are indicated on the *x*-axis. Tetra ac indicates H4K5ac/K8ac/K12ac/K16ac. The *y*-axis indicates the binding ratio (bound fraction per input). Means ± SE (n = 3) and *p* values in a two-tailed Student's *t*-test are shown. *B*, summary of the binding preferences of 21 bromodomain proteins. Two-tailed *p* values are indicated. **$p < 0.01$; *$p < 0.05$.

Supplementary Figure S2

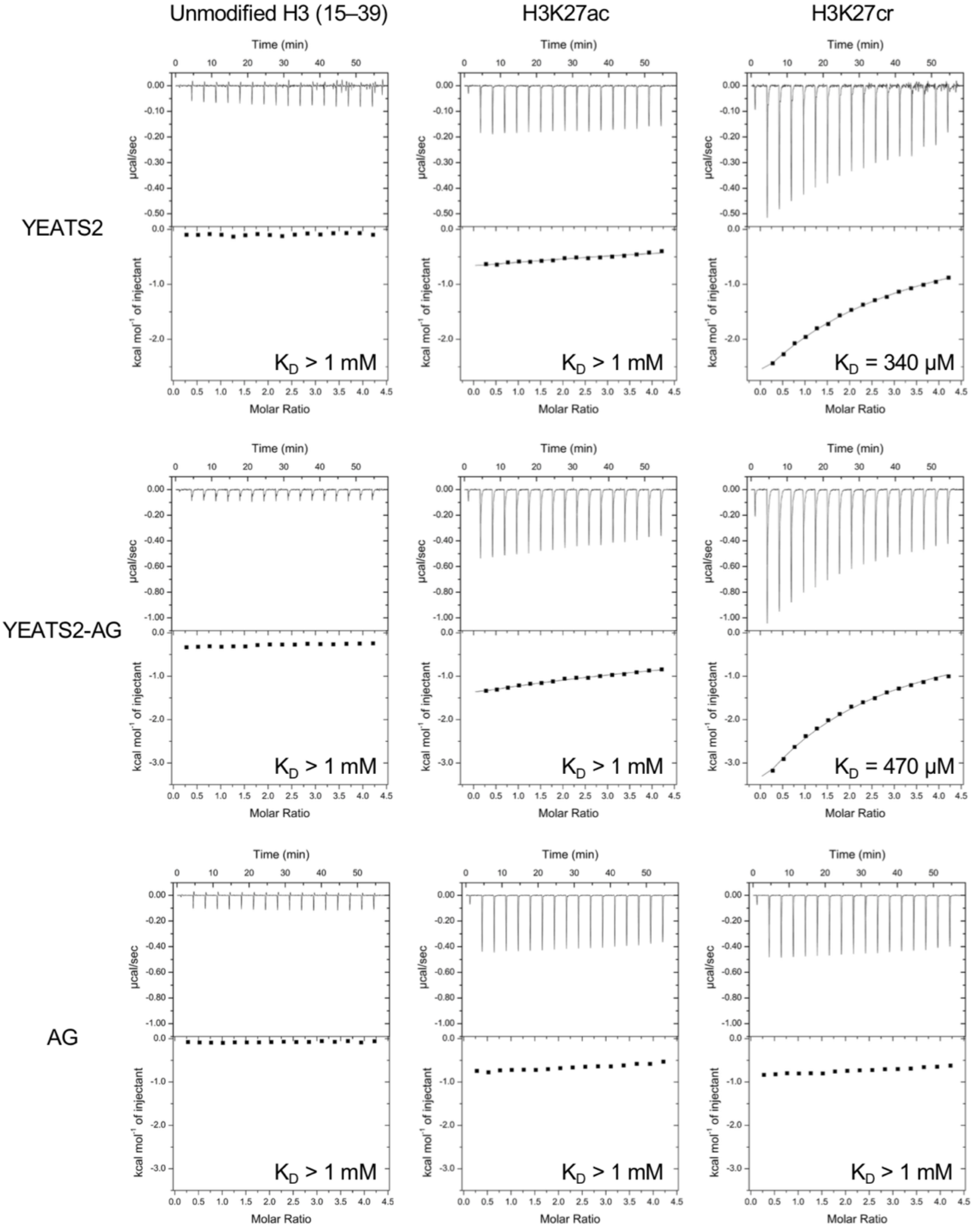

Figure S2. Isothermal titration calorimetry (ITC) analysis of the interaction between the YEATS2-YEATS domain and histone H3 (15–39) peptides. YEATS2, YEATS2-YEATS domain; YEATS2-AG, YEATS2-YEATS domain fused with Azami-Green; AG, Azami-Green. The H3 peptide was either unmodified, acetylated on K27 (K27ac), or crotonylated on K27 (K27cr). In each plot, the top panel is the ITC thermogram and the bottom panel shows its titration curve. K_D, dissociation constant.

Supplementary Figure S3

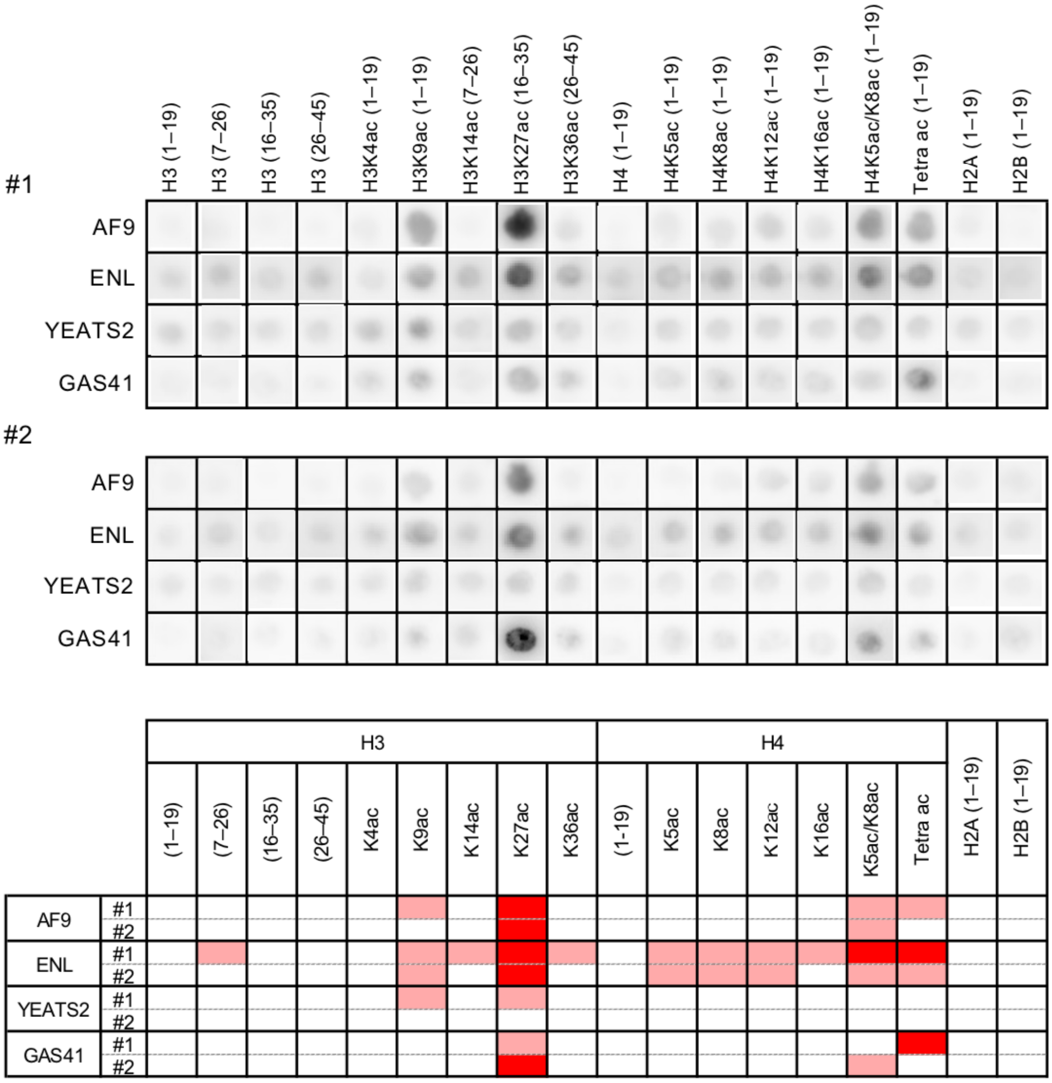

Figure S3. Preferences of YEATS domains determined using the modified histone peptide array. Various peptides are arranged in duplicate on the array glass slide. Representative spots of acetylated modified peptides were selected from two replicate experiments (#1 and #2). Tetra ac indicates H4K5ac/K8ac/K12ac/K16ac. Summary of the signal intensities for each YEATS domain is shown at the bottom. Signal intensities: red, >5 × 10^4; pink, 2.5–5 × 10^4; white, <2.5 × 10^4.

Supplementary Figure S4

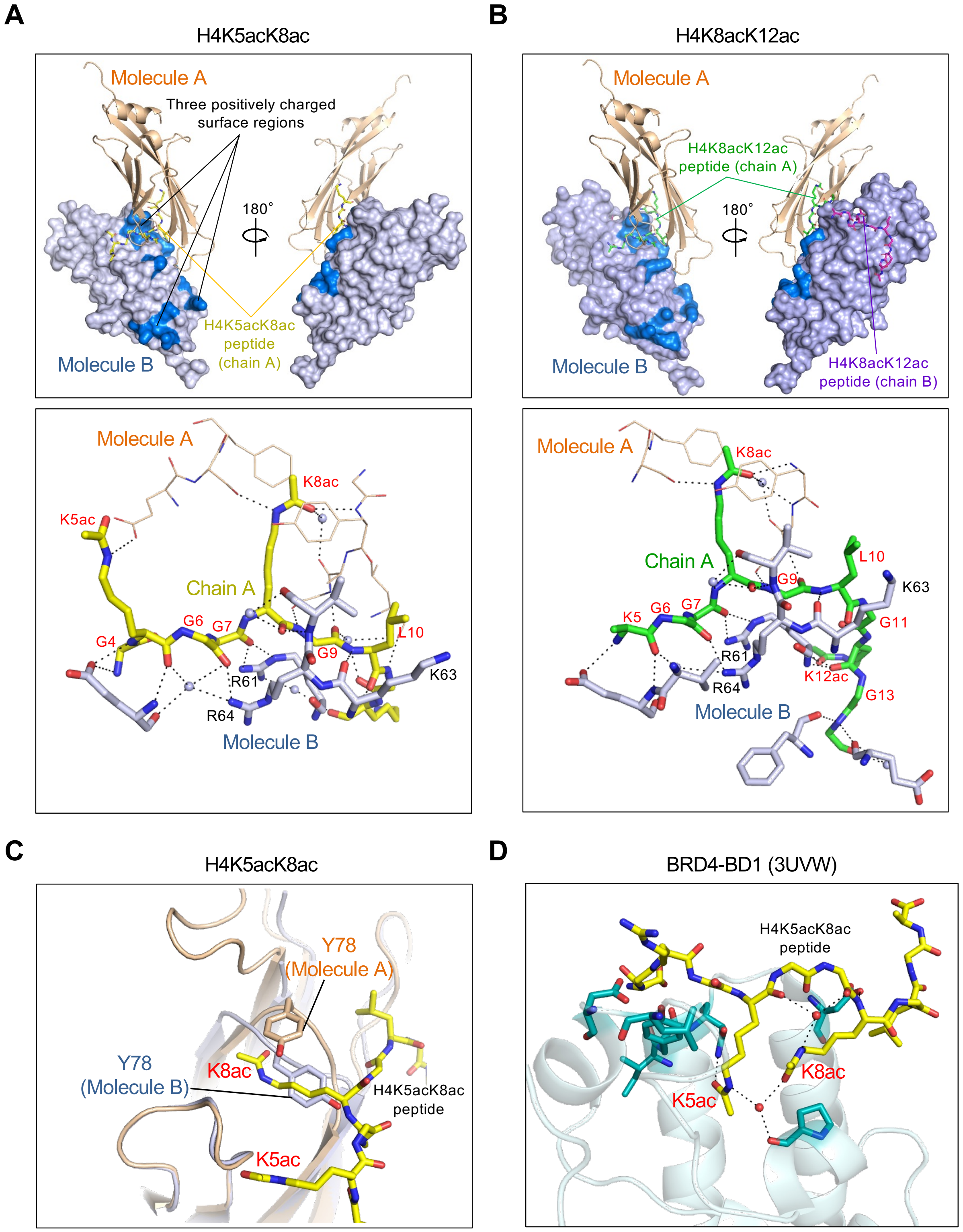

Figure S4. Crystal structures of AF9 in complex with di-acetylated H4 peptides in the asymmetric unit. *A*, *B*, binding modes of the complexes with *A*, H4K5acK8ac peptide (in yellow) and *B*, H4K8acK12ac peptide (in green); the AF9-YEATS molecules A and B are shown in orange and pale

blue, respectively. The bottom panels are close-up views of the hydrogen-bonding networks between the indicated di-acetylated H4 peptides (shown as yellow or green sticks and labeled in red) and the AF9-YEATS symmetric molecule B (depicted as pale blue sticks and labeled in black); hydrogen bonds are shown as black dashes. *C*, superposition of the AF9-YEATS molecules A (orange) and B (pale blue) in the AF9•H4K5acK8ac peptide complex. *D*, close-up view of the first bromodomain of BRD4 (cyan, PDB ID: 3UVW) around the H4K5acK8ac peptide (yellow).

Supplementary Figure S5

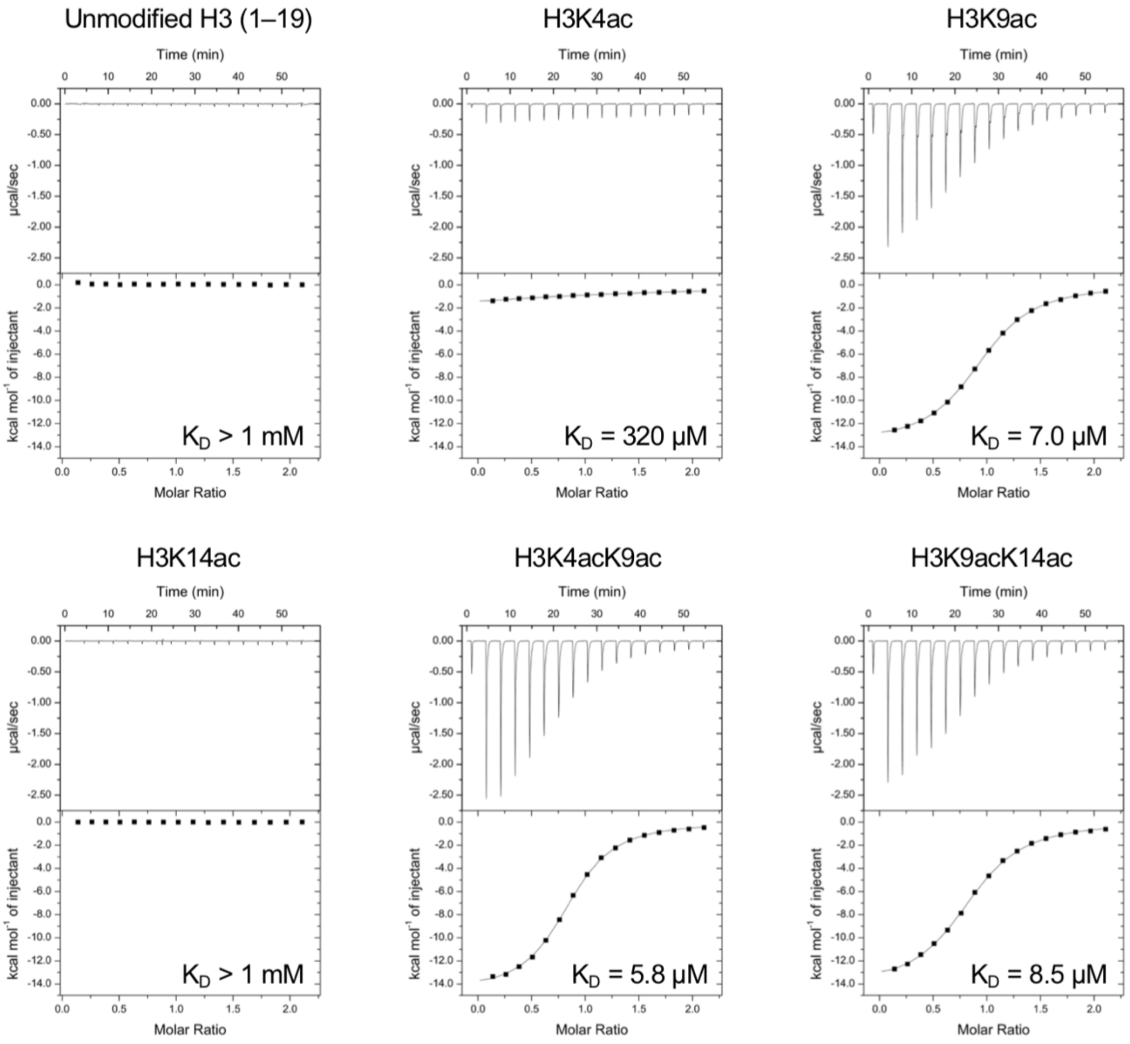

Figure S5. ITC analysis of the interaction between the AF9-YEATS domain and histone H3 (1–19) peptides. Position of lysine acetylation in the H3 peptide is denoted at the top of each panel. The data are shown in the same way as in Figure S2.
